# Modelling adipose tissue-cancer crosstalk: a three-dimensional perspective

**DOI:** 10.1038/s41388-026-03697-w

**Published:** 2026-02-18

**Authors:** Gabriele Strusi, Caterina M. Suelzu, Justin Stebbing

**Affiliations:** Department of Life Sciences, Anglia Ruskin University, Cambridge, UK

**Keywords:** Cancer microenvironment, Experimental organisms

## Abstract

Innovative three-dimensional (3D) systems have become a focus of research due to their ability to better mimic cell-cell and cell-extracellular matrix interactions. Current advances in 3D modelling have the potential to transform pre-clinical research by providing a more biologically relevant recapitulation of the in vivo *cell* environment. Among the published 3D platforms there is a lack of adipose tissue and cancer complex models. Primarily thought to function in triglyceride storage, protection and heat production, adipose tissue is now recognised as a complex and dynamic endocrine organ that secretes factors such as free fatty acids and adipokines, which have been shown to play a role in carcinogenesis. Obesity, a major cause of adipose tissue dysfunction, has also been strongly linked to the development of several types of cancer. 3D model technologies offer an innovative way to investigate adipose tissue-cancer crosstalk by mimicking in vivo conditions. This review aims to present a perspective on the adipose tissue-cancer dynamics and provide an overview of the current 3D models used to reliably reproduce the adipose tissue-cancer interaction in vitro.

## Introduction

The use of new technologies and the research and development of new treatments are increasingly important in cancer and metabolic research, particularly in light of the FDA Modernisation Act 2.0 of December 2022, which aims to address the limitations of traditional 2D systems and to reduce animal use by employing in silico, in chemico, and modern in vitro models [1]. Traditional in vitro 2D cultures do not recapitulate complex structures such as tissues and organs. Kang et al. defined complex in vitro models as all those models that exhibit higher structural complexity than 2D cultures, comprising 3D cultures, organoids, and organs-on-chip [1]. 3D cell culture has thus emerged as a superior system to replicate the in vivo microenvironment with a defined extracellular matrix (ECM) that allows assessment of cell-to-cell interactions [2]. Here, it is thought that the intricate structure of human tissues and organs is often characterised by different cell fate transitions with implications across various diseases [3], making 3D platforms better suited for studying different pathologies.

Adipose tissue has emerged as a key target for advanced experimental models in research, owing to its metabolic and pro-tumorigenic roles [4]. 2D models have proved critical to deepening our understanding of adipose tissue biology while being cost-effective [5]. However, traditional in vitro 2D techniques cannot represent the complexity and dynamism of adipose tissue, which is characterised by three-dimensionality, vasculature, cell-cell and cell-ECM interactions [5]. In vitro 2D systems cannot further support the differentiation of adipose tissue cells as in vivo conditions. Indeed, adipocytes grown in 2D cultures present with small multilocular fat deposits, while in vivo adipocytes display a single lipid droplet that fills most cytoplasm, pushing the nucleus towards the plasma membrane [6]. Therefore, modern 3D models to study adipose tissue as healthy or pathological tissue are required.

Obesity affects white adipose tissue (WAT), which expands via hyperplasia and hypertrophy, with adipocytes increased in number and size, respectively [7, 8]. Obesity is also characterised by low-grade inflammation and a modified cytokine and adipokine secretion and lipolysis of the adipose tissue, leading to a status of systemic inflammation [9, 10]. The altered adipose tissue observed in obesity has been linked to the onset of different diseases, such as type 2 diabetes [11–13] and cancer [14, 15]. Indeed, obesity has been associated with the incidence of different types of cancer, such as breast [16], colon [17], and pancreatic [18] cancer, although the underlying cause has remained elusive.

Cancer is increasingly recognised as a systemic disease, wherein tumour cells undergo metabolic reprogramming and recruit various cell types to sustain tumour mass growth and evade host immune defences [19, 20]. Cancer cells are typically associated with fibroblasts, endothelial cells, and macrophages, which provide structural support, supply nutrients and oxygen, and interact with the immune system [21]. Adipose tissue has a pivotal role in breast cancer, by way of one example, being in direct contact with the mammary gland, while in other types of cancer, such as colorectal and pancreatic cancer, it is thought to interact with the tumour at later stages of the cancer progression, when the tumour leaves the primary site or metastasises [22].

This review aims to describe the current knowledge of the interaction between adipose tissue and cancer cells, with a focus on the 3D human models employed to investigate their crosstalk.

## Adipose tissue

Adipose tissue, also known as fat, is mistakenly thought of as an inert depot with the sole functions of energy storage and body protection. Adipose tissue’s role has been re-evaluated in recent years, as it is now recognised as the largest metabolic and endocrine organ of the human body [23]. This tissue is a type of connective tissue mainly composed of adipocytes or fat cells, which store energy as triglycerides (TGs). Adipocytes arise from the differentiation of mesenchymal stromal cells (MSCs) through a process called adipogenesis, becoming preadipocytes first and mature adipocytes later [24]. Nevertheless, adipocytes represent 90% of the adipose tissue volume, but they account for just about 50% of its cellular composition. Indeed, the non-adipocytic portion, named stromal vascular fraction (SVF), includes multipotent adipose tissue-derived stromal cells (ADSCs), preadipocytes, immune cells (e.g. neutrophils, lymphocytes, macrophages), endothelial cells, and fibroblasts [25]. In adults, about 10% of adipocytes are renewed yearly, with new adipocytes generated via adipogenesis; adipocyte turnover declines with age as a consequence of reduced lipolysis of fat cells, i.e. TG breakdown into free fatty acids (FFAs) and glycerol [26, 27]. A dysregulated adipocyte turnover may also result from metabolic diseases such as obesity and dyslipidaemia. Adipocyte death and clearance, together with progenitor proliferation and adipogenesis, seem to be exacerbated in obese individuals, although the mechanisms that regulate obese fat turnover are not fully understood [28].

### Types of adipose tissue

**Fig. 1:**
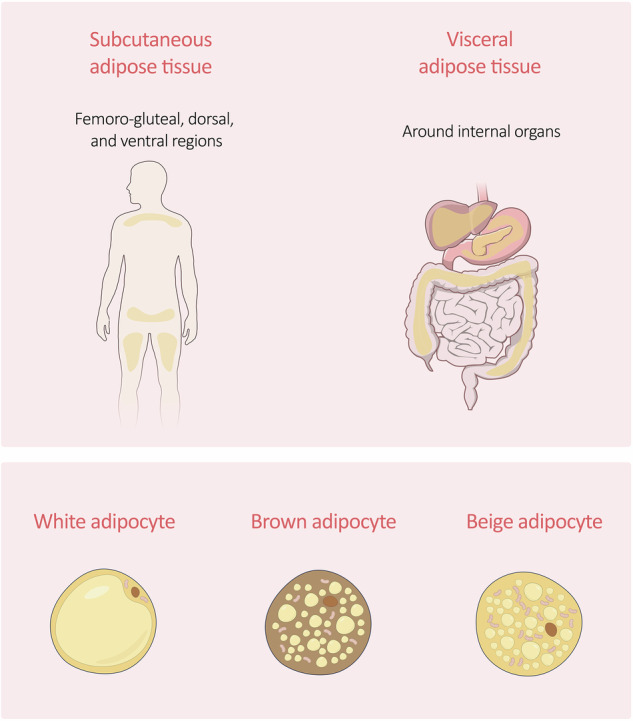
Different types of adipose tissue. Adipose tissue is mainly divided into subcutaneous and visceral. Subcutaneous adipose tissue is found in the femoro-gluteal, dorsal, and ventral regions, while the visceral adipose tissue lines internal organs. At a cellular level, adipose tissue is composed of white, brown, and beige adipocytes, which display different morphologies and functions as determined by their intracellular content (lipid droplets and mitochondria). Their functions range from energy storage (white/beige adipocytes) to heat production and thermogenesis regulation (brown/beige adipocytes).

Adipose tissue is found in different areas of the body and, depending on its location, is described as subcutaneous adipose tissue (SCAT) or visceral adipose tissue (VAT) (Fig. 1). SCAT is mainly located in the femoro-gluteal regions and the dorsal and ventral abdominal regions, representing about 80% of the total body fat [29]. VAT refers to the fat located in the abdominal cavity and surrounding internal organs; it increases with age in both men and women, where it contributes to about 15 and 7% of total body fat, respectively [29, 30]. Here, SCAT adipocytes are described as smaller than VAT adipocytes, being more insulin sensitive and able to prevent ectopic deposition of TG (i.e. deposition of TG in non-adipose tissue cells). Indeed, SCAT serves as the main physiological site to store excess energy. If SCAT energy storage capability is exceeded, fat accumulates in different areas, promoting VAT expansion. The increase in VAT accumulation causes detrimental effects on body health. VAT is highly vascularised and presents with higher levels of glucocorticoid and androgen receptors, together with higher levels of adiponectin if compared with SCAT [29, 31]. Similarly, inflammatory cells infiltrate VAT more than SCAT. Thus, an increase in visceral adiposity leads to an increased production and release of pro-inflammatory cytokines (e.g. tumour necrosis factor α, interleukin-6), which contribute to the systemic inflammation typical of metabolic disorders such as obesity. VAT accumulation is also linked to hyperglycaemia, hyperinsulinemia, and insulin resistance, alongside cardiovascular disease and stroke [29, 32, 33].

At the cellular level, adipose tissue can be further classified as WAT, brown adipose tissue (BAT), and beige or brite adipose tissue (BgAT) (Fig. 1). Adipocyte classification and function are orchestrated by transcription factors and secreted molecules. Key factors include PPARγ, the master adipogenic regulator in all adipocyte types, as well as PGC‑1α, PRDM16, and EBF2, which control mitochondrial biogenesis and thermogenesis in brown and brite adipocytes [34, 35]. Concurrently, adipocytes function as endocrine cells, secreting adipokines and batokines with local and systemic effects. White adipocytes mainly secrete adipokines such as adiponectin, leptin, and resistin, with a role in energy homeostasis, insulin sensitivity, and inflammation. Brown and beige adipocytes release batokines, including Follistatin, BMP7, EPDR1, and SLIT2-C, thus promoting WAT browning and energy expenditure [35]. WAT is the most abundant type of fat tissue, representing about 10% of the total body weight. It is found subcutaneously in the femoral, pubic and abdominal regions and viscerally, surrounding internal organs such as the liver and intestines [36, 37]. WAT adipocytes are spherical in shape, with a single large lipid droplet that forces mitochondria and nucleus to be apically located. The size of WAT adipocytes ranges from 25 to 200 μm, indicating that these cells can expand in response to energy demand and metabolic stimuli. Indeed, their primary function is to store TGs and to release FFAs and glycerol for ATP synthesis in a process called lipolysis [36, 38]. A reduction in white fat cell plasticity has often been associated with metabolic dysregulations [39]. In obese subjects, for example, white adipocytes fail to expand adequately to store excess caloric intake, with the consequent ectopic fat deposition and increased lipotoxicity, which in turn increases the risk of insulin resistance, metabolic disease, and type 2 diabetes [10, 40]. Recent findings demonstrated that WAT consists of distinct adipocyte subpopulations with singular developmental origins and transcriptional profiles. In murine models, Lee et al. showed that a single depot of white adipocytes contains cells with different lipid storage capability, gene expression, and metabolism, named type 1, 2, 3, and undefined adipocytes [41]. Spatial transcriptomic analyses by Bäckdahl et al. revealed mature adipocyte subpopulations within human subcutaneous WAT, termed Adipo^PLIN^, Adipo^LEP^, and Adipo^SAA^. Among them, only Adipo^PLIN^ displayed a transcriptional response to insulin in vivo, thus suggesting that insulin sensitivity depends on the abundance and function of a specific adipocyte subset [42].

BAT is more prominent in foetal and infant life, and it is found in scapular, subscapular, cervical, perirenal, and para-aortic regions [38]. Brown adipocytes present with numerous small lipid droplets and mitochondria, contributing to their brownish colour. Brown adipocytes play a crucial role in the process named non-shivering thermogenesis, by which they metabolise TGs to produce and dissipate heat [43]. Upon fatty acid oxidation, heat will be produced at the expense of ATP via mitochondrial respiration. Furthermore, BAT energy expenditure activity appears to confer protection against body weight gain [38, 44]. Through single-nucleus RNA-sequencing, Sun et al. identified adipocyte subpopulations within human and mouse BAT that differ from the canonical brown and beige lineages. Specifically, they described a rare ALDH1A1+/CYP2E1+ adipocyte subtype that became more abundant at thermoneutral temperatures, able to modulate thermogenesis paracrinally via acetate-mediated signalling, thus limiting energy expenditure. The researchers suggested that this regulatory mechanism could potentially be targeted to fine-tune adipose tissue thermogenic activity [45].

BgAT displays features of both WAT and BAT, developing within subcutaneous WAT; beige adipocytes can either differentiate from preadipocytes or trans-differentiate from white adipocytes [35]. Beige adipocytes exhibit numerous small lipid droplets and many mitochondria, therefore being able to store energy and produce heat. The WAT ‘beiging’ process might be activated by several factors, including diet and exercise, indicating that BgAT can help prevent metabolic dysfunction via energy expenditure [35, 36].

## Adipose tissue 3D modelling

Although murine models continue to be extensively used in research studies, they do not accurately replicate human physiology and/or pathology. Also, the costs associated with animal models, together with ethical considerations and technical feasibility, have further encouraged researchers to develop and employ sophisticated in vitro 3D models. In line with this, recent work has incorporated 3D systems to mimic the structural and functional properties of WAT to elucidate its role in biological processes and dysfunctions, such as obesity [5]. The 3D adipose tissue models here discussed are either scaffold-free, i.e. spheroids and organoids, or scaffold-based (Table 1).

**Table 1:** Research articles reporting 3D models to study the adipose tissue microenvironment.

Authors	Adipose tissue cells	Model	Category
Klingelhutz et al. [47]	Pre-adipocyte cell line, primary SVF cells	Hanging drop	Spheroid
Shen et al. [48]	Primary SVF cells	Low attachment plate	Spheroid
Muller et al. [49]	Primary SVF cells	Stirring incubation	Spheroid
Ioannidou et al. [6]	Primary SVF cells	Low attachment plate	Spheroid
Escudero et al. [51]	Primary SVF cells	GelMA hydrogel	3D scaffold
Pieters et al. [52]	Primary hADSCs	Fibrin-Geltrex^TM^ hydrogel	3D scaffold
Quan et al. [53]	MSCs	Low attachment plate with hydrogel	3D scaffold
Mandl et al. [56]	Primary hADSCs	Low attachment plate	Organoid
Strobel et al. [57]	Primary microvessel fragments, MSCs	Low attachment plate	Organoid

### Spheroids in adipose tissue modelling

Spheroids are 3D cell culture systems that can provide more reliable modelling of in vivo conditions, with more similar tissue organisation, homeostasis, and differentiation. Indeed, differentiation of cells organised in spheroids results in conserved gene expression patterns and tissue organisation [46]. In 2018, Klingelhutz et al. developed a scaffold-free spheroid system generated by employing human SVF cells. Over 35 days of adipogenic differentiation, the spheroids consisted mostly of mature adipocytes and displayed lipid droplet accumulation, as well as greater adiponectin and interleukin-8 secretion when compared with 2D cultures [47]. While the authors proposed using the model for metabolic and pharmacological assays, this system does not fully recapitulate the structural and functional complexity of adipose tissue; the absence of an elaborated ECM, for example, does not support the long-term maintenance of cell types present in the SVF, such as endothelial cells. Similarly, Shen et al. established scaffold-free 3D adipose tissue spheroids by differentiating primary human preadipocytes for 17 days and maintaining them for an additional 25 days [48]. Compared with conventional 2D cultures, the spheroids exhibited mature adipocyte characteristics, including increased lipid accumulation and gene expression profiles closely resembling freshly isolated mature adipocytes, as confirmed by transcriptomic and lipidomic analyses. Functionally, the spheroids responded to insulin and lipolytic stimuli and could tolerate metabolic stress such as fatty acid overload. This model demonstrated long-term stability, as well as compatibility with genetic manipulation, offering a versatile ex vivo system for studying human adipocyte biology and metabolism [48]. Muller et al. employed human SVF cells to originate vascularised adipose tissue spheroids [49]. Along with adipogenic 2D differentiation, they formed the spheroids for 6 days before embedding them in Matrigel^®^. This step supported the survival of endothelial cells and protected the spheroid core from necrosis. Subsequently, the researchers here differentiated the spheroid-forming SVF cells for 17 days to obtain mature adipocytes. Using nude mouse models, they implanted the spheroids of adipose tissue near the subcutaneous inguinal fat pad and interscapular BAT. They demonstrated that most adipocytes within the adipose tissue spheroids closely resembled human mature white adipocytes with a unilocular structure. Furthermore, when transplanted into mouse fat pads, the spheroids showed the capability to connect with the host vasculature. Despite the strengths of this model, the authors used the terms ‘spheroid’ and ‘organoid’ interchangeably when defining the model. Since the vascularised adipose tissue originated from preadipocytes and precursor cells before being embedded within an ECM, the term ‘organoid’ appears more appropriate in this context. Similarly, Ioannidou et al. developed a unilocular vascularised adipose tissue spheroid model using human primary SVF cells; after 6 days, the spheroids were included in Matrigel^®^ and differentiated for up to 40 days [6]. In contrast with the work of Muller et al., this model was characterised after 20, 30, and 40 days and compared with 2D cultures and ECM-free 3D cultures in terms of adipogenic gene expression and adipokine release. Furthermore, Ioannidou et al. added a mixture of lipids to the spheroids to mimic weight gain in vitro. They concluded that the addition of lipids generated ‘obese’ spheroids, with larger unilocular adipocytes and lipid droplets. In this case as well, the model could be referred to as an ‘organoid’.

### 3D scaffolds in adipose tissue modelling

3D scaffolds are widely used models that better resemble the structural organisation of cells in the human body. Several materials can be used to generate the scaffolds, such as natural or synthetic polymers, and their properties can be tuned to obtain different types of support, providing the right microenvironment to the cells of interest [50]. Among the studies here, Escudero et al. generated an innovative 3D model of beige adipose tissue using human WAT-derived SVF cells and ultra-low attachment plates [51]. To promote the formation of vascularised organoids, SVF cells were embedded into photopolymerisable gelatin methacryloyl (GelMA) hydrogels prior to differentiation into brown adipocytes for 21 days. This group suggested that the model used provides a valuable tool for the investigation of beige adiposity studies and metabolic diseases, such as obesity and type 2 diabetes. Pieters et al. combined human ADSCs (hADSCs) and fibrin-Geltrex^TM^ hydrogel and differentiated the progenitor cells into mature adipocytes for 14 days [52]. They also stimulated the adipocyte scaffold with either oleic or palmitic acid to obtain obese-like adipocytes with increased lipid droplet size. This particular study showed that the obese 3D system increased basal lipolysis, impaired insulin sensitivity, and significantly upregulated the expression of the interleukin-6 gene in macrophages treated with obese scaffold supernatant. Thus, they concluded that the model represents a useful tool for studying the mechanisms underlying adipocyte pathophysiology. The study published by Quan et al. explored the therapeutic potential of beige adipose tissue organoids developed from MSCs, adipose tissue-derived microvascular fragments, and adipose acellular matrix hydrogel [53]. Beige adipogenic differentiation occurred over 21 days, after which organoids displayed enhanced metabolic activity and improved metabolic dysfunction when implanted into the interscapular region of type 2 diabetic and obese nude mice, mitigating liver steatosis, promoting thermogenesis of BAT, and reducing white adipocyte size. Overall, their model could serve as a platform to investigate possible therapeutic strategies for the amelioration of adipose tissue-related metabolic dysfunctions.

### Organoids in adipose tissue modelling

Organoids are typically defined as 3D miniatures composed of a self-organised cluster of cells that mimic the structure and function of an organ. Generally, organoids are generated by pluripotent stem cells or progenitor cells because of their capacity to differentiate into the type of cells that will compose the so-called ‘mini-organ’ [54, 55]. Adipose tissue organoids have been recently included in research papers, with the aim of creating unique models that better resemble human adipose tissue. For example, Mandl et al. employed ADSCs to generate adipose tissue organoids by means of ultra-low attachment plates [56]. ADSCs were initially seeded to form spheroids and subsequently treated for 18 days to differentiate into mature adipocytes, whose adipogenic marker (i.e. FABP4, PPARγ2, Adiponectin, C/EBPβ) expression increased with culture time. However, the organoids produced lacked an ECM support and microvasculature, thus partially reproducing adipose tissue structure and functions. In contrast, Strobel et al. produced a more complex and functional organoid by mixing together human-derived microvessel fragments and MSC-derived preadipocytes prior to embedding in a collagen matrix [57]. After 7 days of culture, organoids exhibited vascular sprouts and lipid-containing adipocytes. Moreover, adipogenic gene expression, insulin receptor expression, and interleukin-6 secretion were upregulated upon microvessel inclusion in the organoid. The authors suggest that the model can represent a robust platform for both research and therapeutic applications.

## Cancer and adipose tissue crosstalk

**Fig. 2:**
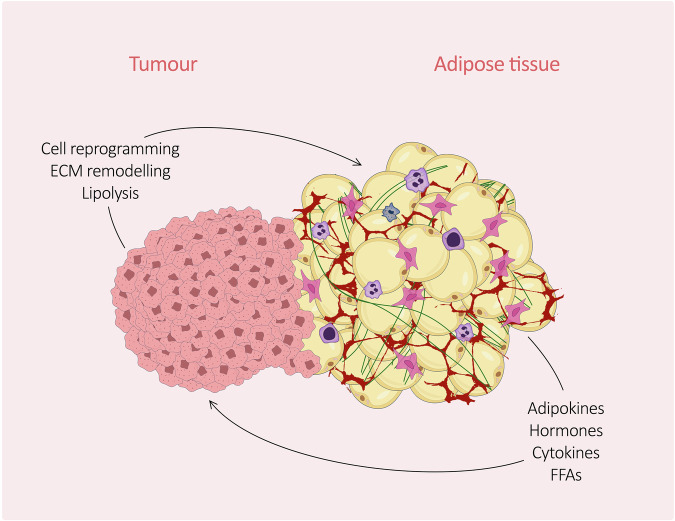
Crosstalk between adipose tissue and cancer. A mutual influence between tumour cells and adipocytes is the core of the complex dynamics in the tumour and adipose tissue relationship. Cancer cells reprogramme adjacent cells such as adipocytes [85], immune cells (in blue and purple) [86], fibroblasts (in pink) [87], and endothelial cells (vessels in red) to support their proliferation [88] and induce lipolysis to fuel their own growth [62]. In turn, adipocytes release adipokines, hormones, cytokines, and FFAs to support tumour growth [4].

In light of the complexity of adipose tissue as an endocrine organ, its role in cancer development and progression attracted interest. Adipose tissue secretes factors, provides nutrients, and modifies the surrounding ECM [4], thus affecting tumour growth (Fig. 2). Cancer cells can interact with adipose tissue, and the existence of a bidirectional interchange of adipokines and lipids between WAT and cancer has been reported [21]. Tumour cells have the ability to reprogramme their metabolism to adapt to the surrounding environment and to alter the cell fate of other cell types to support their growth. For example, upon recruitment, fibroblasts shown to acquire cancer-associated fibroblast (CAF) characteristics [21]; similarly, adipocytes can be reprogrammed towards a cancer-associated phenotype [22]. In turn, factors released by adipocytes can alter the expression of genes related to cancer development [58].

### Systemic effect of adipose tissue on cancer

Due to the endocrine functionality of adipose tissue, both cytokines and the adipokines produced can have a systemic effect on tumour cells. Indeed, the circulating levels of adiponectin and leptin were reported to have a prognostic value for several cancers [4]. Furthermore, cytokines such as TNFα, IL-6, IGF-1, and CXCL12 have been identified as promoters of cancer progression [59, 60]. Also, circulating adipose-derived exosomes have shown to reduce programmed cell death in colorectal cancer and provide chemotherapy resistance [61]. Cancer-associated adipocytes have been demonstrated to overexpress pro-inflammatory cytokines and to increase their lipolysis [22], providing fuel for tumour growth. In states of adipose tissue dysfunction, FFAs released or not internalised by adipocytes can be exploited by cancer cells as fuel, together with higher circulating insulin and glucose [22].

### Paracrine effects of adipose tissue on cancer

Some cancers develop and grow adjacent to adipose tissue, such as breast, pancreatic, kidney, melanoma, and prostate cancer, as aforementioned [4]. Other tumours interact with adipose tissue at later stages, once the tumour has spread from the primary site to neighbouring tissues [22]. It has been shown that breast cancer cells can elicit lipolysis and fatty acid transfer to increase their own proliferation and migration, and this was also observed in a 3D model of breast adipose tissue [62, 63]. Furthermore, tumour cells are able to induce dedifferentiation of adipocytes towards other cell types (i.e. myofibroblasts, macrophage-like cells) to support tumour growth [64]. Cancer cells can induce changes in CAFs to release lactate and pyruvate, which are used to fuel tumour progression [65]. Here, cancer-recruited fibroblasts can also deeply change the ECM through integrin-mediated signalling to facilitate tumour growth [66].

### Obese adipose tissue and cancer

As previously mentioned, obesity can deeply alter the adipose tissue microenvironment, impacting also cancer development and progression. There is a clear link between obesity and the development of several types of cancer, as shown in a data linkage cohort study with a span of 22 years, which highlighted a strong association between obesity and 13 types of cancer [67]. Similarly, Petrelli et al.’s meta-analysis highlighted the poor survival outcome in patients with obesity and breast, colon, and uterine cancer [68]. Cancer cells have proved to exploit adipose tissue dysfunction; indeed, dysfunctional adipose tissue can lead to insulin resistance, which can reduce adipocytes’ lipid internalisation and increase lipolysis, fuelling cancer cells with circulating FFAs, insulin, and glucose [4]. When adipose tissue increases in size in obese conditions, the ECM is remodelled to make space for larger adipocytes, deeply changing its core structure [69]. ECM alterations include, inter alia, a rearrangement of the stromal components with the recruitment of other cell types such as CAFs and M2 macrophages, thus contributing to the development of a pro-tumorigenic environment [69]. The ECM deposition from the different recruited cell types, together with the accumulation of basement membrane components (i.e. collagens, laminins, fibronectins) increase the overall ECM stiffness [69]. Indeed, Chen et al. showed that obese SCAT overexpresses several collagen genes [70], as well as laminin [71].

As the microenvironment of adipose tissue has such a central role, it is relevant to consider and include it in 3D models for the study of the fat-cancer intricate relationship. Adipocytes and their secreted lipids and factors, ECM proteins to support and create the environment in which tumour masses develop and grow, are only a few examples of the players that must be part of a comprehensive 3D model. The following section discusses the most recent human 3D models of adipose tissue and cancer.

## Cancer and adipose tissue 3D modelling

**Fig. 3:**
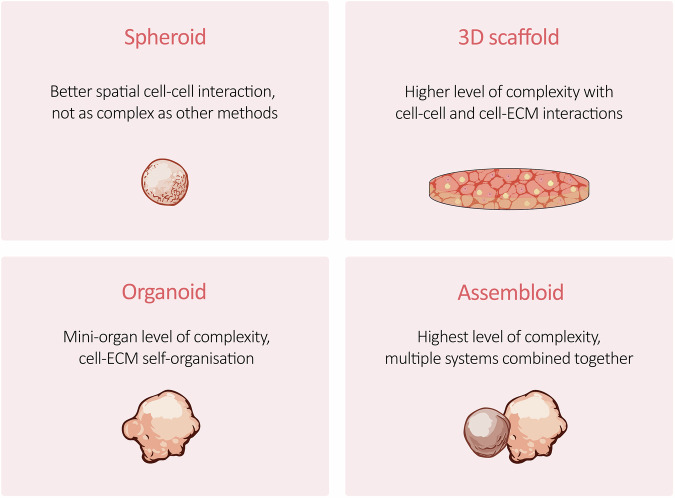
Overview of 3D models of adipose tissue and cancer crosstalk. The figure depicts an overview of the 3D models employed by researchers to recapitulate the adipose tissue-cancer dynamics in vitro. *Spheroids* are aggregates of cells that self-organise in spherical structures when placed in appropriate physical conditions (i.e. low attachment vessel, hanging drop, agitation, microfluidics) [46]. Spheroids can be mono- or multi-cellular and formed with or without the addition of a structural protein or a mixture of them (i.e. collagen, Matrigel^®^). *3D scaffolds* are structures that support the growth of cells, mimicking the natural extracellular matrix (ECM). The ECM has a fundamental role in providing physical and chemical signals to support cell growth and maintenance [50]. Scaffolds can be made of natural or synthetic polymers, and their structural properties can be tuned to adapt to specific applications. *Organoids* are self-organising 3D in vitro models that reproduce some of the hallmarks of real organs at a millimetre scale [89]. The specialised cell types (i.e. pluripotent stem cells, progenitor cells, mature cells) self-organise by forming structures that resemble full-scale organs or regions of organs [90]. Organoids can be used for a wide range of applications, from developmental studies to modelling disease and drug discovery. *Assembloids* are the result of multiple organoids/spheroids combination to integrate different systems and study their interactions [90, 91]. Assembloids are widely used in neuroscience studies, where different regions of the brain are merged to study neural interactions. This 3D model is also acquiring more attention in cancer research, offering the possibility to study the tumour microenvironment effect on cancer-related mechanisms such as metastasis cell dissemination [92–94].

2D monolayer cultures of cancer cells are routinely used to test the effect of new and existing molecules and compounds of interest, including drugs, making 2D cultures the most used in vitro pre-clinical models. This methodology has been shown to be flawed, as the results in most cases do not accurately reflect the effect of the same compounds on patients [72]. An important parameter to consider is the difference in complexity between a 2D cell monolayer, which does not fully recapitulate cell-cell and cell-ECM interactions, and a 3D structure such as a living tissue [73]. For this reason, models employing cancer cells organised in a 3D fashion are attracting increasing attention as valuable tools for cancer research. In the last 10 years, several approaches have been explored to model the interaction between adipose tissue and cancer, with most of the work focusing on breast cancer. The models here discussed and summarised in Table 2 can be categorised into spheroids, 3D scaffolds, organoids, and assembloids as depicted in Fig. 3.

**Table 2:** Research articles reporting 3D models to study the adipose tissue-cancer interaction.

Authors	Adipose tissue cells	Cancer cells	Method	Category	Cancer type	Crosstalk analyses
Seo et al. [74]	Primary hADSCs	WiDr, PC3, MCF7	Pluronic-coated PDMS	Spheroid	Colon, prostate, breast	Microscopy, condition media effect, protein expression, in vivo xenograft
Paré et al. [75]	Primary hADSCs	MCF7	Agarose moulds	Spheroid and cells	Breast	Microscopy, gene and protein expression
Goto et al. [76]	Primary hADSCs	PDX	Low attachment plates	Spheroid	Breast	Microscopy, gene expression, ELISA, in vivo xenograft
Ritter et al. [77]	Primary hADSCs	MCF7, MDA-MB-231, MDA-MB-361	Hanging drop	Spheroid	Breast	Microscopy, gene expression, ELISA, transcriptomics
Horder et al. [78]	Primary hADSCs	MDA-MB-231	Agarose moulds, hydrogel	3D scaffold	Breast	Microscopy, gene expression
Delort et al. [79]	Primary hADSCs	MCF7, MDA-MB-231	Dermal substrate	3D scaffold	Breast	Gene expression
Bessot et al. [80]	Primary BM-MSCs, SGBS	LNCaP, C4-2B	Hydrogel	3D scaffold	Prostate	Microscopy, Gene expression, ELISA, metabolic activity
Rebeaud et al. [81]	Primary adipocytes	MDA-MB-231	Fibrin matrix	3D scaffold/Transwell	Breast	Microscopy, lipolysis assay
Mertz et al. [82]	Primary SVF cells	MCF7, MDA-MB-231	Hanging drop	Organoid	Breast	Microscopy
Lei et al. [84]	3T3-L1 fibroblasts	MCF7	Hanging drop	Assembloids	Breast	Microscopy

### Spheroids in adipose-cancer modelling

Spheroids represent the second most commonly used method in modelling human adipose tissue and cancer interaction among the studies analysed. Seo et al. employed heterogeneous spheroids to study the relationship between adipose tissue and three different cancers, i.e. colon, prostatic, and breast cancer, showing how cancer epithelial-to-mesenchymal transition can be influenced by lipids released by adjacent adipose tissue [74]. Cancer cells and hADSCs were mixed in different ratios and then seeded onto a cell-repellent polymeric chip instead of the canonical cell-repellent plastic culture plates. The authors concluded that the byproducts of the cancer-associated adipocytes were responsible for a migration-favourable microenvironment. It was not mentioned how the hADSCs were differentiated into adipocytes for the experiments. While cancer spheroids appear round and well-formed, heterogeneous spheroids do not look as round and complete. One factor to consider with 3D cultures is the repeatability and reproducibility of the results, requiring a standardised process to obtain comparable structures throughout the repeated experiments. Paré et al. used agarose-coated plates to form mammospheres composed of MCF7 [75]. The main conclusion was that the cancer cells are able to modify the adipocytes’ phenotype through the secretion of adrenomedullin. The model used can be considered only partially 3D as they co-cultured the mammospheres of breast cancer cells with 2D monolayers of ADSCs. Goto et al. employed ADSCs isolated from the adipose tissue of breast cancer patients and cancer cells from a murine patient-derived xenograft to form spheroids on low attachment plates [76]. They showed that the adipokine adipsin was responsible for an adipose-epithelial cell interaction capable of enhancing breast cancer cells’ stemness properties. The ADSCs were not differentiated prior to the formation of the 3D culture; therefore, the secreted adipokine profile is not comparable to that of adipose tissue in in vivo conditions. A further spheroid model was also published by Ritter et al., who studied the effect of undifferentiated hADSCs on breast cancer cells in heterogeneous spheroids, generated with the hanging drop method [77]. Here, hADSCs were shown to influence breast cancer cells with the activation of more cancer-invasion-related genes. In particular, hADSCs derived from obese patients were found to promote the transcription of stemness-related genes. Spheroid models are structurally more intricate than 2D monolayer cultures, yet their level of complexity is not comparable to other 3D models that better recapitulate the in vivo tissue architecture.

### 3D scaffolds in adipose-cancer modelling

3D scaffolds are the most cited model that focuses on studying the adipose tissue-cancer interaction. Most of the work here employed 2D cells embedded in a 3D matrix, with only one study describing embedded pre-formed spheroids into the 3D structure. Indeed, Horder et al. used agarose moulds to originate spheroids using primary hADSCs, which were then bioprinted into the hyaluronic acid-based hydrogels [78]. Subsequently, spheroids inside the hydrogel constructs were differentiated to obtain mature adipocytes and the hydrogels containing breast cancer cells were laid on top. They concluded that the interaction observed was similar to the expected interaction between breast cancer cells and adipose tissue. Delort et al. employed a dermal substrate initially seeded with fibroblasts and preadipocytes, and then different breast cancer cell lines, keratinocytes, or MCF10A cells were applied over [79]. They observed that the cancer cell lines were able to induce dedifferentiation of the preadipocytes compared to the keratinocyte control. Bessot et al. showed that the crosstalk between adipocytes and prostate cancer cells leads to a deregulated lipid metabolism with implications in therapy resistance [80]. They used two different types of adipocyte progenitor cells, bone marrow MSCs (BM-MSCs) and Simpson-Golabi-Behmel syndrome (SGBS) cells, which were embedded in a hydrogel prior to being subjected to adipogenic differentiation. In this work, cancer hydrogels were generated by encapsulating spheroids of LNCaP or C4-2B cells and then co-cultured with the adipose tissue hydrogels, although the spheroid formation protocol used is not described [80]. Rebeaud et al. utilised primary adipocytes embedded in a 3D fibrin matrix and then co-cultured the 3D system with breast cancer cells growing in 2D, via a transwell system [81]. The authors showed that the fibrin matrix was able to sustain the integrity of adipocytes for 5 days, while adipocytes grown in 2D rapidly lose their viability. The transwell system allowed co-culture of the adipocytes with the breast cancer cells and the observation of a crosstalk increase during obesity; nevertheless, the method presented is not structured enough to mimic in vivo conditions, especially when compared with the other 3D models presented. Overall, 3D scaffolds are effective models that partially recapitulate in vivo conditions with many available options and the possibility to finely tune the material’s structural properties.

### Organoids in adipose-cancer modelling

To date, only one research article mentioning organoids of adipose tissue and cancer cells is available. Mertz et al. used primary SVF cells and breast cancer cell lines to develop the so-called geometrically inverted mammary organoids, where an outline of epithelial cells surrounds an adipose core, ultimately invaded by cancer cells [82]. The researchers highlighted how such a model may enable the understanding of adipose tissue-driven cancer cell invasion through the epithelium. However, the model does not resemble native tissue, in which the epithelium of the mammary ductal lumen, a possible site for breast cancer development, is surrounded by stroma and adipose tissue. Well-designed organoids can better mimic in vivo structural complexity and can be applied to cancer research to further investigate the adipose-cancer dynamics.

### Assembloids in adipose-cancer modelling

Assembloids are generally defined as models that combine two or more organoids or spheroids to generate a highly elaborate model. Different techniques can be used to form assembloids, and these are comprehensively discussed in Zhu et al.’s work [83]. Assembloid models have emerged recently, and only one research article combining assembloids, adipose tissue, and cancer could be found. Recently, Lei et al. published a study that evaluates how adipose tissue influences breast cancer cell invasion. By using a wettability-patterned microchip and the hanging drop method, they produced spheroids of mature adipocytes and spheroids of breast cancer cells. After that, they merged the spheroids in a single drop to allow the formation of the assembloids, which were subsequently transferred into a collagen hydrogel [84]. Interestingly, the authors did not use primary human adipocytes to generate the adipose tissue spheroids, but differentiated 3T3-L1 fibroblasts. It was suggested that the mechanical compression at the junction where fat and tumour spheroids merged was responsible for adipocyte dedifferentiation and reprogramming into myofibroblasts, with the latter promoting integrin α-5-driven invasion of breast cancer cells. Assembloids are promising research tools that could refine 3D models in the future, with more accurate reproduction of in vivo conditions. In line with this, we are currently developing an assembloid system in our laboratory to investigate interactions between adipose tissue and different types of highly invasive tumour cells, with the aim of complementing, combining, and extending existing 3D modelling approaches towards the study of fat-cancer crosstalk.

## Conclusions and perspectives

Adipose tissue 3D models have proven a more effective approach to investigate fat functions and dynamics, as well as its interaction with other cell types, including cancer cells. This review explored the most relevant studies involving adipose tissue 3D systems cultured either alone or in combination with cancer cells, highlighting the relevance of these models and their need to further understand the pathophysiology of adipose tissue and its involvement in cancer development and progression. 3D spheroids are the cheapest, easiest to reproduce, and high-throughput systems, providing the best solution for studying basic interactions and drug screening. Spheroids offer the lowest complexity with limited heterogeneity and tumour microenvironment representation capabilities, thus not fully resembling in vivo conditions. 3D scaffolds are the most used model for studying mechanobiology, with tunable structural properties, allowing the specific analysis of physical interactions between cells and ECM. 3D scaffolds are more complex than spheroids, facilitating the study of a more heterogeneous tumour microenvironment. Their complexity results in poor scalability for high-throughput analysis, also depending on the effect of the biomaterial used. Organoids have a higher complexity with different cell types that self-organise, resembling in vivo tissues. They can be cultured long-term and expanded from specific patients’ tissues. Organoids lack complete vascularisation and the presence of the immune system. Assembloids present with the highest level of complexity, allowing the fusion of multiple systems to study cell signalling, crosstalk, and drug efficiency. These platforms display a more heterogeneous tumour microenvironment, although the greatest complexity is associated with the advanced technical expertise required, higher costs, and issues in reproducibility and scalability.

To summarise, there is a lack of high-quality data, and current models still present several main limitations, such as the absence of an appropriate ECM, absent or incomplete vascularisation, the use of non-mature adipocytes, and the absence of immune components. The costs involved, along with the advanced techniques required for analysis and the expertise needed to manage these models, in particular assembloids, make them less accessible. Overall, the development of advanced models that more effectively combine adipose tissue and cancer cells is necessary to demonstrate their predictive accuracy. The generation of such models would provide an innovative platform for mechanistic studies on fat-cancer crosstalk, drug screening, and the development of therapeutic strategies.
